# A preparation method for the highly permeable ceramic microfiltration membrane – precursor film firing method

**DOI:** 10.1039/c7ra12314k

**Published:** 2018-01-12

**Authors:** Xiaoqin Yin, Kang Guan, Peng Gao, Cheng Peng, Jianqing Wu

**Affiliations:** School of Materials Science and Engineering, South China University of Technology Guangzhou 510640 China imjqwu@scut.edu.cn +86 2087110273 +86 2087111669

## Abstract

A method called the precursor film firing method is proposed to improve the permeance of ceramic microfiltration membranes by avoiding intermediate layer and dip-coating process, and efficiently control the thickness of the separation layer. In this method a precursor film is prepared independently of the support by a film coating machine. This precursor film consists of two layers: Al_2_O_3_/PVA layer made of Al_2_O_3_ powder and polyvinyl alcohol (PVA), and polyvinyl butyral (PVB) layer. The precursor film is pasted on the support and fired to obtain membranes. Because the intermediate layer and dip-coating process are avoided in this method, the fabricated membranes show high permeance. For the fabricated membrane with average pore size = 0.18 μm and separation layer thickness = 10.7 μm, the permeance is 3890 L m^−2^ h^−1^ bar^−1^. Also the separation layer thickness can be controlled efficiently. And the precursor film can be used on fabricate curved membranes such as cylindrical membranes.

## Abstract 2

A new technique is proposed to improve the permeance of ceramic membranes.

## Graphical abstract

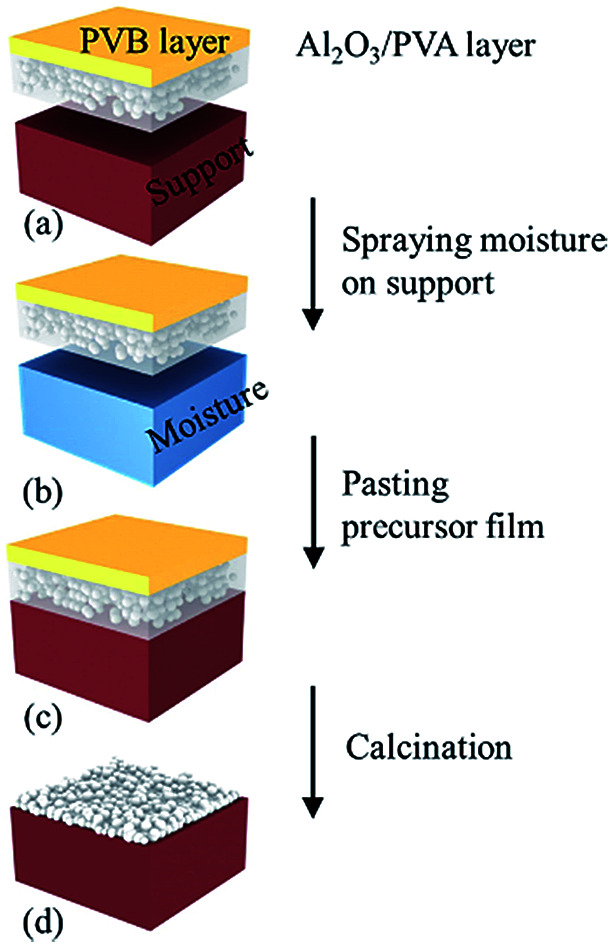

## 1. Introduction

In comparison with other membranes, such as polymeric membranes, ceramic membranes show higher cost because of their high sintering temperatures and raw materials. However ceramic membranes normally show considerable mechanical strength, thermal stability and chemical compatibility.^1,2^ Due to these properties, they are promising in some filtration processes under harsh conditions, such as high pressure and high temperatures.

Ceramic microfiltration membranes normally consist of three layers: support layer, intermediate layer and separation layer.^3,4^ The support layer provides mechanical strength, and the separation layer gives selectivity efficiency.^4^ The intermediate layer that bridges the support layer and the separation layer plays several roles:^3^ usually the precursor system of the separation layer such as ceramic slurry^5–11^ and sol^12–15^ are coated on supports by dip-coating technique. In the absence of the intermediate layer, the small particles in the precursor system for the separation layer will probably penetrate to the large pores of the support and decrease the permeance of the membrane. Moreover, coating a separation layer directly on a support with large pores normally may make the separation layer peel off easily.^3^ However, the intermediate layer will increase the total thickness of membrane system, which will reduce the permeance of membrane. Moreover, the fabrication of the intermediate layer will increase the total fabrication costs of membrane system. Hence, the ideal ceramic microfiltration membrane should consist of a support with large pores and a very thin and defect-free separation layer with controllable pore sizes.^3^

The permeance of membrane is sensitive to the thickness of the separation layer. The separation layer of commercial microfiltration membranes is generally in the range of 10–20 μm.^3^ Controlling the thickness of the separation layer is an important issue in the membrane fabrication process. In traditional dip-coating process, the liquid precursor is absorbed towards the substrate under the driving force of capillary suction pressure of the support or the external applied pressure.^5^ The coating thickness of the separation layer is affected by many parameters, including viscosity, density and surface tension of the precursor system; porosity, surface roughness and radius of the support; the contact time; and the withdrawal speed, *etc.*^5^ Hence, it is complex to control the thickness of the separation layer by adjusting so many parameters in traditional dip-coating technique.

Recently, Qin *et al.*^16,17^ attempted to fabricate membranes without intermediate layer based on one-step coating method and sacrificial-interlayer method. The obtained membranes show relatively high permeance in comparison with the membranes fabricated using traditional dip-coating process. However, because the dip-coating technique was also used in these two methods,^16,17^ the small particles in separation layer might penetrate to the big pores of support. There is still some room to improve these two methods. Moreover it is inconvenient to control the separation layer thickness using these two methods.

This study aims to propose a novel ceramic microfiltration membrane preparation method to improve the permeance of membrane by avoiding intermediate layer and dip-coating process, and efficiently control the separation layer thickness. In this method a precursor film is prepared independently of the supports by a film coating machine. This precursor film consists of two layers: Al_2_O_3_/PVA layer made of Al_2_O_3_ powder and polyvinyl alcohol (PVA), and polyvinyl butyral (PVB) layer. The precursor film is pasted on the support and fired to obtain membrane with high permeance. Because of the important role of precursor film in this method, this novel method is called precursor film firing method. It is noted that the pore size of microfiltration membrane is in a relatively wide range: 0.05–10 μm.^4^ To illustrate the mechanism of precursor film firing method, this study will concentrate on the membranes with pore size around 0.1–0.5 μm. However the applicability of the proposed precursor film firing method is not limited in this pore size range.

## 2. Experimental

### 2.1 Materials

#### 2.1.1 For the fabrication of supports

Commercially available α-Al_2_O_3_ powder with mean particle size = 9 μm (99.5% purity, Nanjing Tansail Advanced Materials Co., Ltd., China) was used as main material. Polyvinyl alcohol (PVA) (1799, Shanghai Aladdin Biochemical Technology Co., Ltd., China) was used as polymeric binder. Ethylene imine polymer (PEI) (Shanghai Aladdin Biochemical Technology Co., Ltd., China) and polyacrylic acid (PAA) (solid mass fraction = 30%, Tianjin Kermel Chemical Reagent Co., Ltd., China) were added as dispersant. Rice starch (Dongguan Rilong, Co. Ltd., China) was used as pore-forming agent. Boehmite (JR14, Al_2_O_3_ mass fraction = 75%, Xuancheng Jingrui New Materials Co., Ltd., China) was used as sintering aid. Glacial acetic acid (CH_3_COOH) (99.5% purity, Chinasun Specialty Products Co., Ltd, Jiangsu, China) was used to balance the pH value of support slurry. Deionized water was used as solvent.

#### 2.1.2 For the fabrication of precursor films

Commercially available α-Al_2_O_3_ powder with mean particle size = 0.52 μm (99.5% purity, Showa Denko Co., Ltd., Japan) was used as main material. PVA (1799, Shanghai Aladdin Biochemical Technology Co., Ltd., China) was used as polymeric binder. PAA (solid mass fraction = 30%, Tianjin Kermel Chemical Reagent Co., Ltd., China) was used as dispersant. Silicone-based defoamer (Nanjing Qicheng New Type Material Co., Ltd, China) was used to reduce air bubbles. Fluorocarbon surfactant (Capstone FS-60, DuPont, USA) was used to reduce the surface tension of membrane slurry. Deionized water was used as solvent for membrane slurry. Polyvinyl butyral (PVB) powder (Shanghai Aladdin Biochemical Technology Co., Ltd., China) was used as main raw material for preparing PVB layer. Ethanol (99.9% purity, Tianjin Fuyu Huagong Co., Ltd, China) was used as solvent for dissolving PVB powder.

### 2.2 Fabrication of supports

Qin *et al.*^18^ lowered the sintering temperature for fabricating high-purity porous alumina support by using boehmite. Similar to Qin *et al.*,^18^ the present study also used boehmite as a sintering additive to fabricate the supports. There are three main steps.

#### 2.2.1 Preparation of α-Al_2_O_3_ slurry

200 g PVA solution (PVA mass content = 2%), 180 g α-Al_2_O_3_ powder (*D*_50_ = 9 μm), 45 g rice starch and 1.8 g PAA were mixed evenly. Meanwhile, the pH of the solution was kept at approximately 5.0 for achieving the electrostatic stabilization.

#### 2.2.2 Preparation of boehmite sol

12 g boehmite and 0.6 g PEI were added in 150 mL deionized water while stirring. The pH of the mixture was also adjusted to around 5.0 for achieving the electrostatic stabilization.

#### 2.2.3 Preparation of support

The prepared α-Al_2_O_3_ slurry was gradually added into the prepared boehmite sol by titration and stirring. After drying and sieving, the powders were placed in a disk-shaped module with diameter of 27.0 mm and thickness of 3.5 mm. The green support body was obtained by applying 50 MPa pressure on the disk-shaped module. The green support body was then sintered in air with the schedule that from room temperature to 600 °C with heating rate = 2 °C min^−1^ and soaked for 1 hour, subsequently heated at 10 °C min^−1^ to 1550 °C and soaked for 3 hours. After being sintered at 1550 °C for 1 hour and polished by diamond polishing paste (45 μm), the supports were obtained.

### 2.3 Fabrication of microfiltration membranes using precursor film firing method

#### 2.3.1 Preparation of Al_2_O_3_/PVA slurry and PVB solution

PVA solution was obtained by dissolving 2.4 g PVA in 21.6 g deionized water. Then, 10.2 g α-Al_2_O_3_ powder (*D*_50_ = 0.52 μm), 25.8 g deionised water, 0.36 g PAA, 0.12 g silicone-based defoamer and 0.18 g fluorocarbon surfactant were mixed in a ball mill for 15 minutes. After that, the prepared PVA solution was added in the ball mill and mixed for another 30 minutes. The air bubbles were removed from the Al_2_O_3_/PVA slurry with a vacuum pump. PVB solution was obtained by dissolving 5 g PVB powder in 95 g ethanol.

#### 2.3.2 Preparation of precursor film

**Fig. 1:**
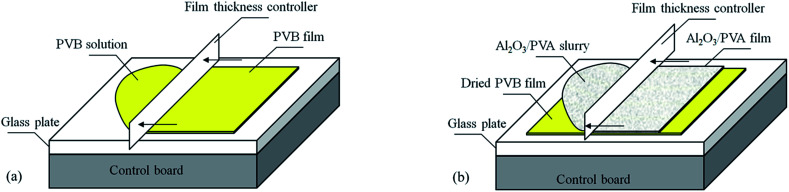
Schematic representation of the preparation of the precursor film (a) fabrication of PVB film (b) fabrication of Al_2_O_3_/PVA film.

As shown in Fig. 1a, the PVB film was prepared by coating the PVB solution on a glass plate using a film coating machine (Automatic Film Application, BEVS Industrial Co., Ltd., China). The thickness of the PVB film was kept at approximately 15 μm with a film thickness controller (the accuracy of this film thickness controller = ±2 μm). After that, the PVB film was dried at room temperature for 30 minutes. As shown in Fig. 1b, the Al_2_O_3_/PVA slurry was then coated on the dried PVB film with the film coating machine. Using the film thickness controller, the thickness of the Al_2_O_3_/PVA layer was also controlled to be about 15 μm, 20 μm, 25 μm, 30 μm, 35 μm and 40 μm, respectively. The Al_2_O_3_/PVA layer was dried at room temperature for 12 hours. Subsequently, the precursor film that consists of the Al_2_O_3_/PVA layer and the PVB layer was separated from the glass plate.

#### 2.3.3 Coating the precursor film on the support

**Fig. 2:**
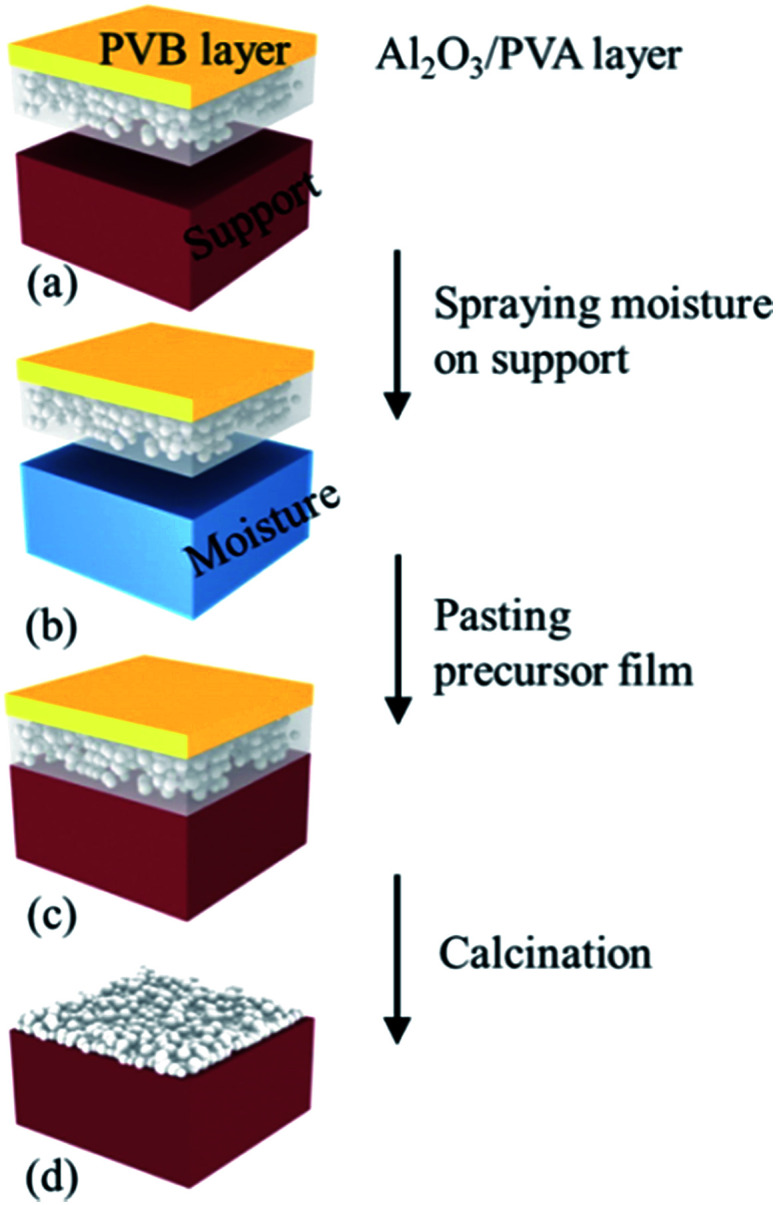
Schematic representation of fabricating ceramic microfiltration membranes using the precursor film firing method.

Soluble polymer is often mixed in inorganic particle suspension to provide mechanical strength and adhesiveness to particulate coatings.^19^ In the precursor film firing method, PVA was used as a binder to pasting the precursor film and the support. The precursor film (Al_2_O_3_/PVA and PVB composite film) was coated on the support as schematically shown in Fig. 2. The support was moistened (Fig. 2a and b) using water spray.

Then, the precursor film was pasted on the support (Fig. 2b and c). When the film contacted the wet surface of the support the PVA in the Al_2_O_3_/PVA layer would partially become wetting, which provided an adhesive force for pasting the precursor film on the support. After drying at room temperature for 24 hours the precursor film with the support was calcined in air with the schedule that from room temperature to 600 °C with heating rate = 2 °C min^−1^ and soaked for 1 hour, subsequently heated at 5 °C min^−1^ to 1280 °C and soaked for 1 hour. During the calcination process PVA, PVB and other organic components in the precursor film were oxidized and volatilized. The remaining Al_2_O_3_ was sintered and the separation layer was formed (Fig. 2d).

### 2.4 Characterizations

The surface tension of the Al_2_O_3_/PVA slurry was measured using a contact angle measuring instrument (FM40Mk2 Easy Drop, KRÜSS GmbH, Germany) at 25 °C. The morphology of the Al_2_O_3_/PVA film was observed using a stereo microscope (L330-M1000, AOSVI, China). The thickness of the membrane and the morphology of the membrane and the support were determined with a scanning electron microscope (SEM) (ZEISS EVO 18, Germany). The pore size distribution and the porosity of the support were tested applying a mercury porosimetry (MIP) (Micrometrics AutoPore 9510, USA). The contact angle of mercury for MIP was set as 130°.

Water treatment like drinking water product, municipal wastewater treatment, and food and beverage industries is an important application of microfiltration membranes.^1^ The present study concentrates on measuring the water permeance of membranes. The water permeance of membrane and support was measured using a fully automated fluid and gas handling systems (OSMO Inspector 2.0, Poseidon, Convergence, Netherlands) at 25 °C. As the pressure of the deionized water was gradually increased, the corresponding flow through the membrane was recorded. The water permeance was calculated based on the relationship between the transmembrane pressure and the flow. To avoid non-stationary transient effects, the membrane and the support were saturated with deionized water before the pressure was applied. The pore size distribution, largest pore size, and average pore size of membranes were tested and calculated according to ASTM: F316-03.^20^ The flexural strength of support is determined by three-point bending test according to ASTM: C1161-13.^21^ In three-point bending test, the support is cut into rectangle-shaped bar with size of 26 (length) × 4 (width) × 3 (thickness) mm^3^. The span of three-point bending test is fixed at 20 mm.

## 3. Results and discussions

### 3.1 Performance of the support

**Fig. 3:**
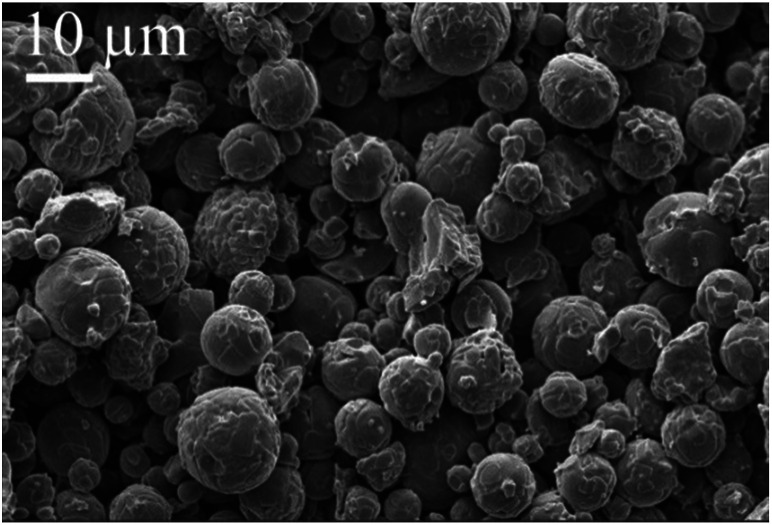
Morphology of the support.

Table 1 lists the flexural strength of supports in this work and literature. The strength of ceramic supports are normally related to the porosity and the pore size. The support in this work shows higher strength than that in Qin *et al.*^18^ This is because the support in this work has smaller porosity than that in Qin *et al.*^18^ The support in this work shows lower strength than that in Dong *et al.*^22^ This is probably because the support in this work has larger average pore size than that in Dong *et al.*^22^Fig. 3 shows the morphology of the support surface viewed using SEM. It can be seen that the Al_2_O_3_ grains are spherical and with the size of around 10 μm.

**Table 1:** Flexural strength of supports in this work and literature

Category	Average pore size (μm)	Porosity	Flexural strength (MPa)	Reference
α-Al_2_O_3_	4.6	0.31	61 ± 3	This work
α-Al_2_O_3_	4.7	0.51–0.52	39–41	Qin *et al.*^18^
α-Al_2_O_3_	3.0	0.39	87 ± 2	Dong *et al.*^22^

**Fig. 4:**
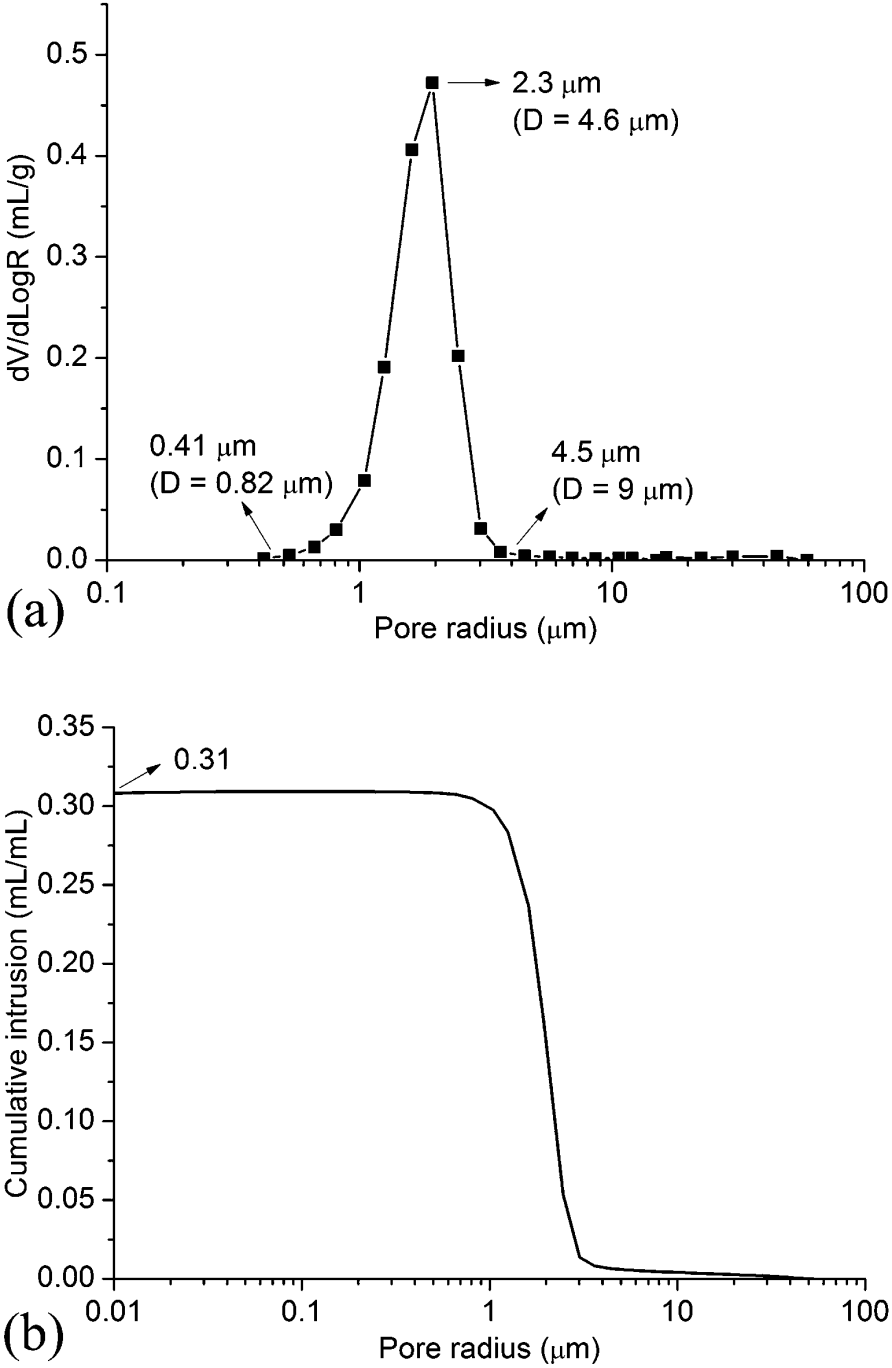
Pore size distribution and porosity of the support. (*D* = pore diameter) (a) pore size distribution (b) porosity.

Fig. 4 shows the pore size distribution and the porosity of the support measured using MIP. As shown in Fig. 4a, the pore diameter ranges from 0.82 μm to 9 μm, and the mean diameter is approximate 4.6 μm. The support shows a narrow pore size distribution (Fig. 4a) and the porosity of the support is 0.31 (Fig. 4b).

**Fig. 5:**
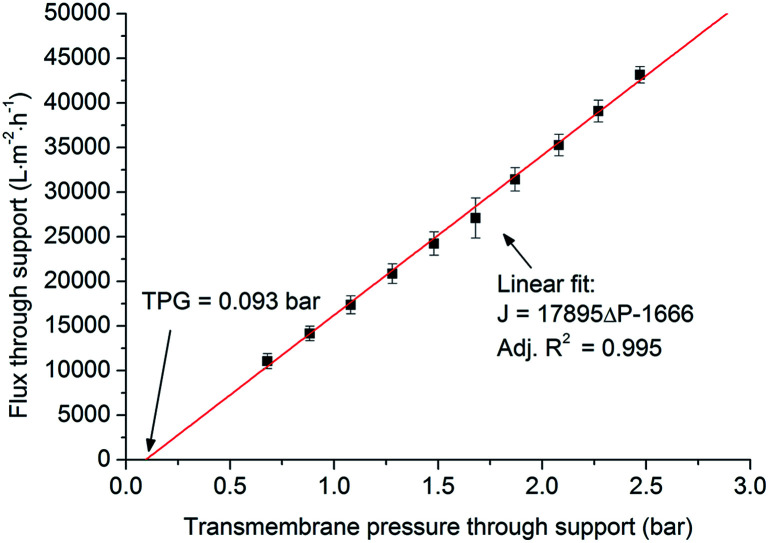
Flux *versus* transmembrane pressure for the support (TPG = threshold pressure gradient).

As shown in Fig. 5, the flux and the transmembrane pressure through the support exhibit a linear relationship (eqn (1)), which is similar to Darcy's law for the flow through a porous material (eqn (2)).^12,23–25^ The slope (17 895 L m^−2^ h^−1^ bar^−1^) in eqn (1) represents the water permeance of the support. With *J* = 0 L m^−2^ h^−1^, eqn (1) gives Δ*P* = 0.093 bar. This pressure (0.093 bar) is called threshold pressure gradient (TPG). In some cases for the low-velocity flow through a porous material, the flow might not occur until the transmembrane pressure increases to the value of TPG.^26,27^

1 *J* = 17895Δ*P* − 1666

where, *J* is the flux through support (L m^−2^ h^−1^), Δ*P* is the transmembrane pressure through support (bar).2
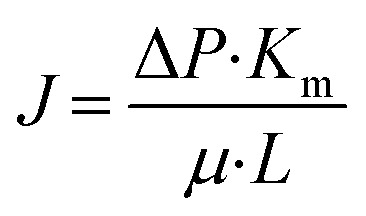
where, *K*_m_ is the permeability of the membrane (m^2^), *μ* is the fluid viscosity (Pa s), *L* is the support thickness (m), Δ*P* is the transmembrane pressure (Pa).

Table 2 lists the water permeance of the supports for microfiltration membrane in this work and literature. The water permeance of support in this work shows same order of magnitude in comparison with the water permeance of supports in literature. In general the average pore size, the porosity and the support thickness are important factors which influence the water permeance. The support in this work shows similar average pore size, porosity and support thickness to that in Dong *et al.*^8^ Accordingly, the support in this work shows similar water permeance to that in Dong *et al.*^8^

**Table 2:** Water permeance of supports in this work and literature

Category	Average pore size (μm)	Porosity (%)	Thickness (mm)	Permeance (L m^−2^ h^−1^ bar^−1^)	Reference
α-Al_2_O_3_	4.6	0.31	3.5	17.9 × 10^3^	This work
Natural zeolite	5.9	0.35	3.0	26.6 × 10^3^	Dong *et al.*^8^
Rutile	2.1	0.43	—	10.4 × 10^3^	Wang *et al.*^10^
α-Al_2_O_3_	2.2	0.46	4.7	7.6 × 10^3^	Qin *et al.*^16^

### 3.2 Influence of surfactant on the formulation of Al_2_O_3_/PVA film

**Fig. 6:**
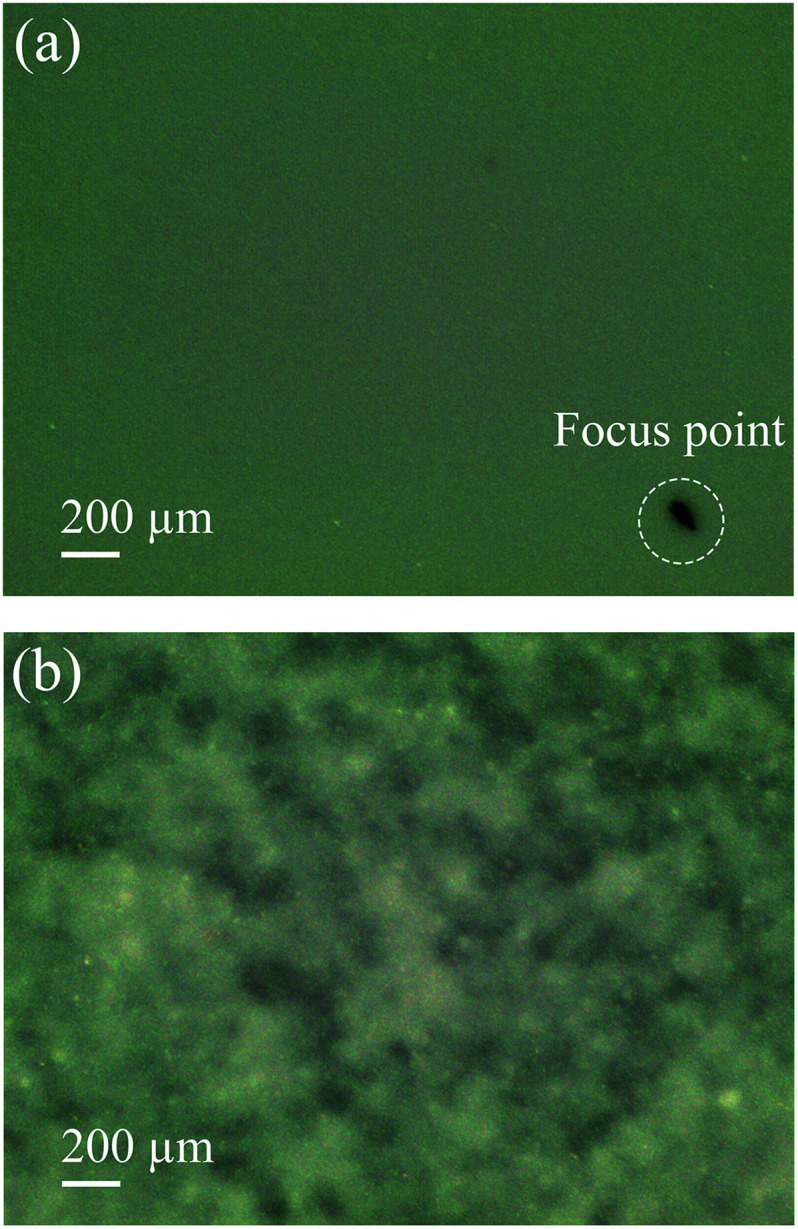
Stereo microscope photos of the Al_2_O_3_/PVA slurry coated on the PVB film with and without fluorocarbon surfactant (thickness of the Al_2_O_3_/PVA film is about 20 μm; magnification times of the stereo microscope = 25).

As shown in Fig. 6, if the surfactant is added in the Al_2_O_3_/PVA slurry a smooth Al_2_O_3_/PVA film can be obtained (Fig. 6a), while if the surfactant is not added in the Al_2_O_3_/PVA slurry the prepared film is uneven (Fig. 6b). This is because the addition of surfactant changes the surface tension of the Al_2_O_3_/PVA slurry. As a result the viscosity and the dispersion degree of Al_2_O_3_ powder are affected which makes the alumina powder disperse more evenly in the composite. The measured surface tensions of the Al_2_O_3_/PVA slurry made with and without fluorocarbon surfactant are 50.3 mN m^−1^ and 76.2 mN m^−1^, respectively. Due to the relatively high surface tension, the Al_2_O_3_/PVA slurry without surfactant was uneven and the Al_2_O_3_/PVA film could not be formed smoothly. Hence, the film prepared using Al_2_O_3_/PVA slurry with a relatively low surface tension is preferred.

### 3.3 Function of the PVB layer

**Fig. 7:**
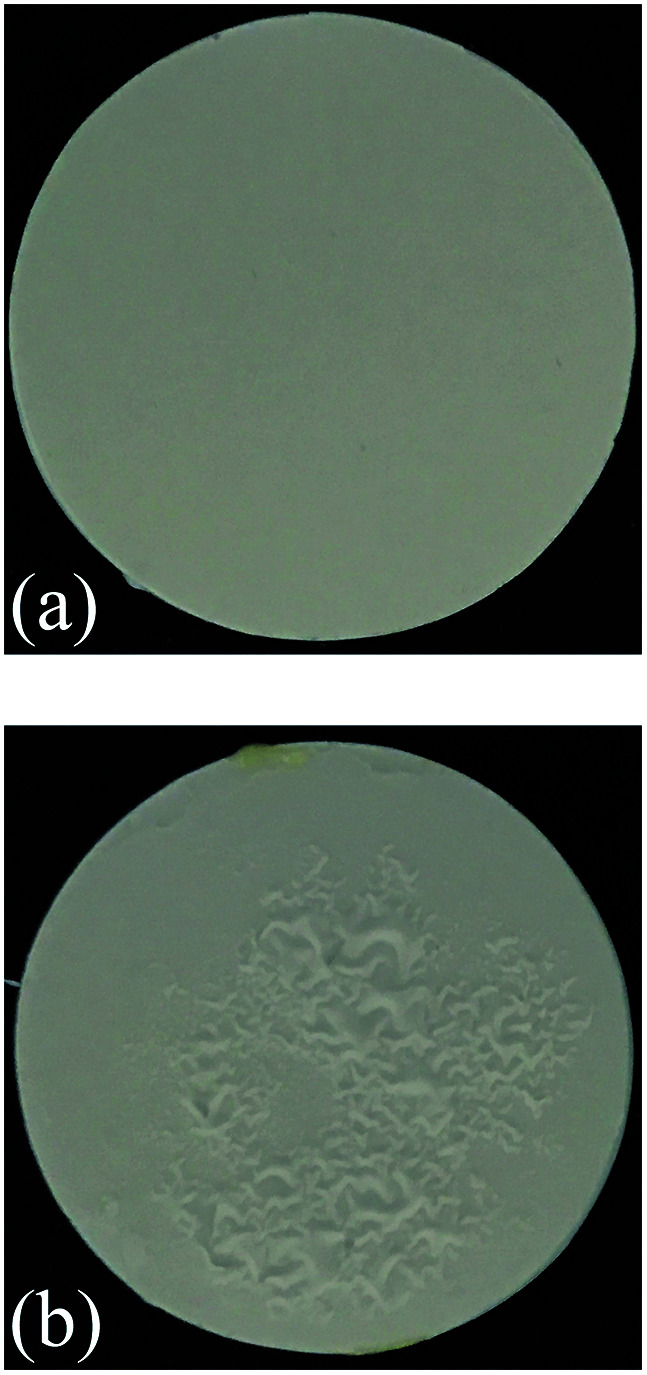
Photos of the membranes fabricated with and without PVB layer (thickness of the Al_2_O_3_/PVA layer of the precursor film is about 25 μm) (a) with PVB layer (b) without PVB layer.

The precursor film consists of Al_2_O_3_/PVA layer and PVB layer. The Al_2_O_3_/PVA layer is calcined as the separation layer of membrane, while the PVB layer is used to inhibit the possible crinkles in the precursor film when the precursor film is pasted on the support. As mentioned in Section 2.3, during the process of pasting the precursor film on the support, the Al_2_O_3_/PVA layer of the precursor film will absorb the moisture on the support surface. The PVA in the Al_2_O_3_/PVA layer will partially become wetting, which provides an adhesive force for pasting the precursor film on the support. However during this process the PVA will also become swelling. As a result, crinkles might form in the precursor film. After calcination, the crinkles will be more severe. Because the PVB layer has good toughness and ductility, and is insoluble in water, it can provide a horizontal restraining force to inhibit the formation of crinkles. Fig. 7 shows that the membrane fabricated with PVB layer exhibits no significant defects (Fig. 7a), and the membrane fabricated without PVB layer has obvious crinkles (Fig. 7b).

### 3.4 Performance of the membrane

#### 3.4.1 Morphology

**Fig. 8:**
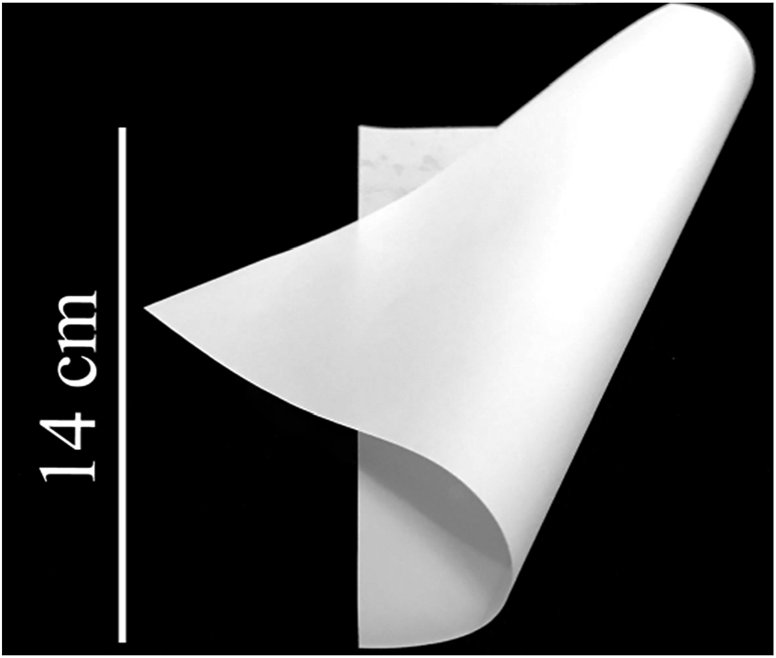
Photo of the precursor film (thickness of the Al_2_O_3_/PVA layer of the precursor film is about 25 μm).

Fig. 8 is the photo of the precursor film (Al_2_O_3_/PVA and PVB composite film). The precursor film is flexible. The flexibility of the precursor film mainly depends on the PVA in the Al_2_O_3_/PVA layer and the PVB layer, because both the PVB and the PVA are flexible. The flexibility of the precursor film provides a potential for applying the precursor film firing method in fabricating curved membranes *e.g.* cylindrical membranes in addition to flat membranes.

**Fig. 9:**
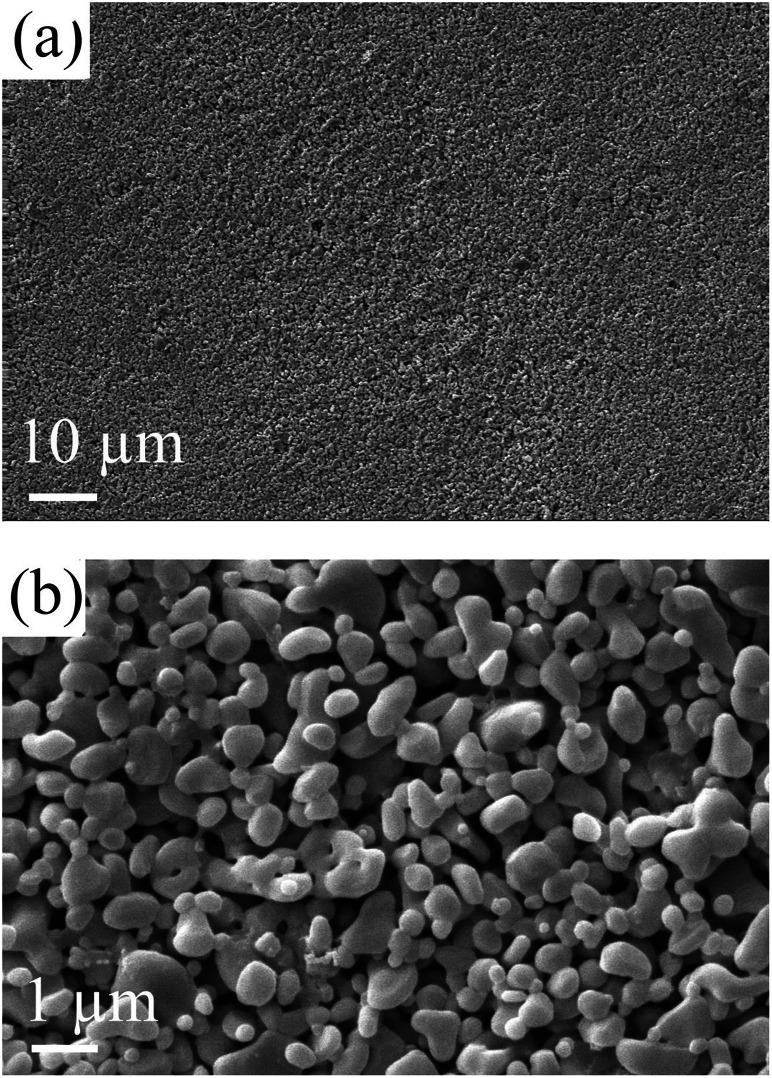
Morphology of the membrane fabricated with the precursor film firing method (thickness of the Al_2_O_3_/PVA layer of the precursor film is about 25 μm).

Fig. 9 shows the morphology of the membrane surface after calcination. The membrane is smooth and no significant defects can be observed.

#### 3.4.2 Pore size distribution

**Fig. 10:**
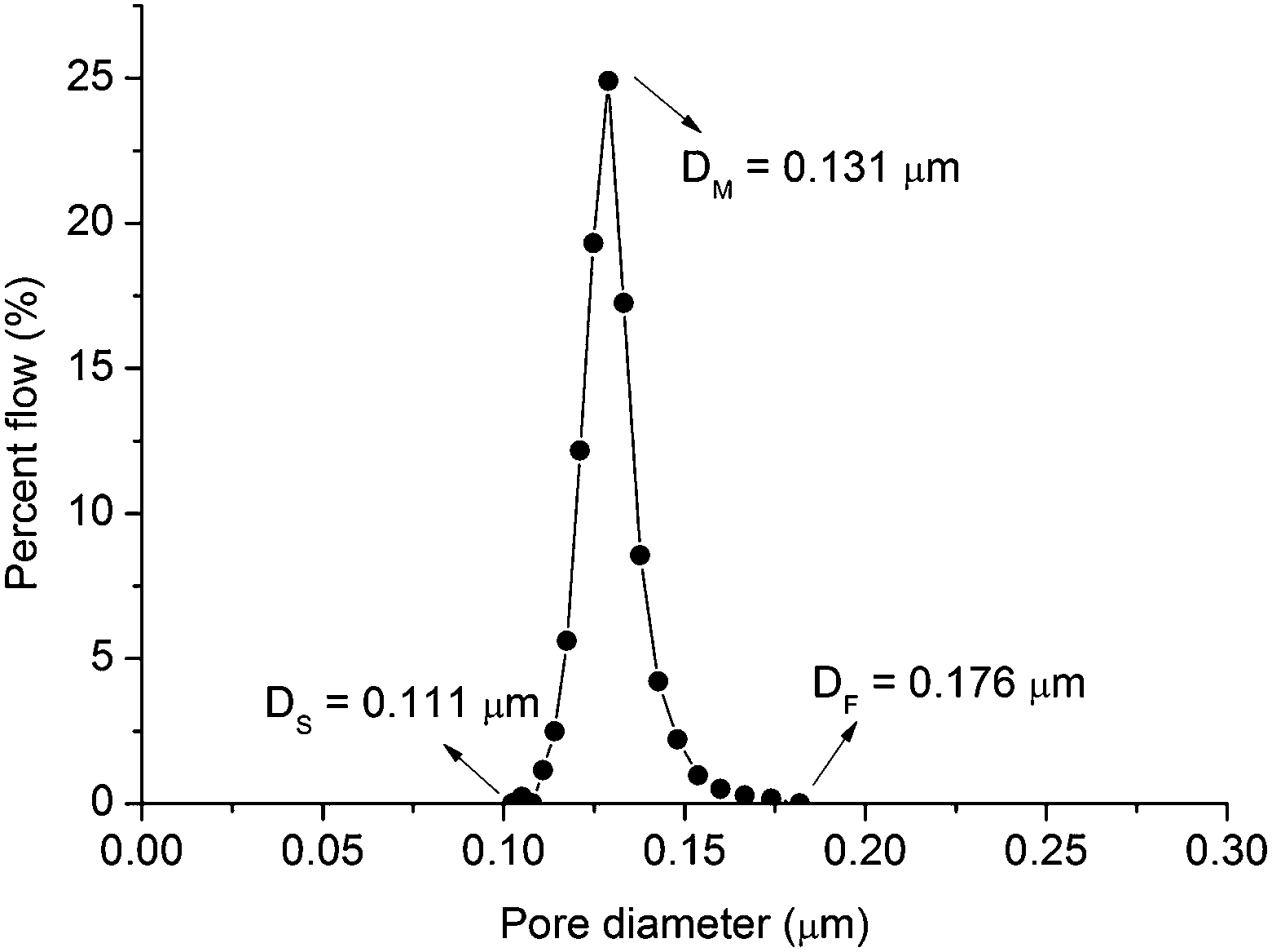
Pore size distribution of membrane fabricated with the precursor film firing method (thickness of the Al_2_O_3_/PVA layer of the precursor film is about 25 μm) note: *D*_s_ = diameter of smallest pore; *D*_M_ = diameter of mean flow pore; *D*_F_ = diameter of maximum pore.

The pore size distributions of the membranes were measured and determined according to ASTM: F316-03.^20^Fig. 10 shows the measured pore size distribution of the membrane. The membrane shows a narrow pore size distribution, which is beneficial to improve the selectivity efficiency.

#### 3.4.3 The thickness of the separation layer

**Fig. 11:**
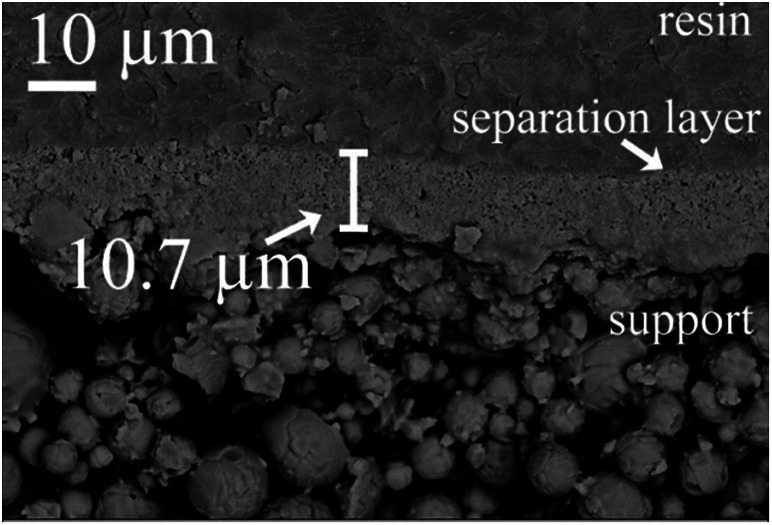
Cross section of membrane (thickness of the Al_2_O_3_/PVA layer of the precursor film is about 15 μm).

Fig. 11 shows the cross section of the membrane (thickness of the Al_2_O_3_/PVA layer of the precursor film is around 15 μm). The small particles in the separation layer do not penetrate to the pores of the support.

**Fig. 12:**
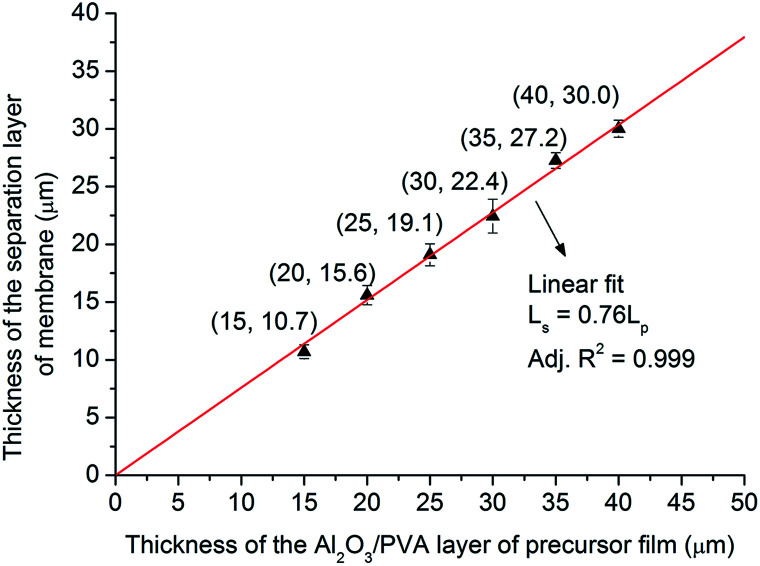
Thickness of the separation layer of membrane *versus* thickness of the Al_2_O_3_/PVA layer of precursor film.

Fig. 12 shows the relationship between the thickness of the separation layer and the thickness of the Al_2_O_3_/PVA layer of the precursor film. With increasing the Al_2_O_3_/PVA layer thickness from 20 μm to 40 μm, the separation layer thickness increases from 10.7 μm to 30 μm. The separation layer of commercial microfiltration membranes is in normally in the range of 10–20 μm.^5^ In other words, the thickness of the separation layer fabricated with the precursor film firing method meets the commercial requirement. In addition, it is found that the thickness of the separation layer (*L*_s_, μm) linearly increases with the thickness of the Al_2_O_3_/PVA layer of the precursor film (*L*_p_, μm) (eqn (3)). The gradient in eqn (3) represents the volume deformation of Al_2_O_3_/PVA layer during the drying at room temperature and calcination at 1280 °C.

3 *L*_s_ = 0.76*L*_p_

where, *L*_s_ is the thickness of the separation layer [μm], *L*_p_ is the thickness of the Al_2_O_3_/PVA layer of the precursor film (μm).

This linear relationship illustrates that it will be convenient to control the thickness of the separation layer with the precursor film firing method proposed in this study. As mentioned earlier, the thickness of the separation layer prepared by the traditional dip-coating method is affected by a variety of parameters, including viscosity, density and surface tension of the suspension; porosity, surface roughness and radius of the support; the contact time; and the withdrawal speed, *etc.*^5^ Hence it will be complex and difficult to control the thickness by adjusting so many parameters.

From the viewpoint of controlling the thickness of the separation layer, the precursor film firing method proposed in this study is more attractive than the traditional dip-coating technique.

#### 3.4.4 Water permeance

The water permeance of membrane is calculated as:

4 *P*_M_ = *J*/Δ*P*

where, *P*_M_ = the water permeance of membrane, L m^−2^ h^−1^ bar^−1^; *J* = the flux through the membrane, L m^−2^ h^−1^; Δ*P* = the transmembrane pressure through the membrane, bar.

**Fig. 13:**
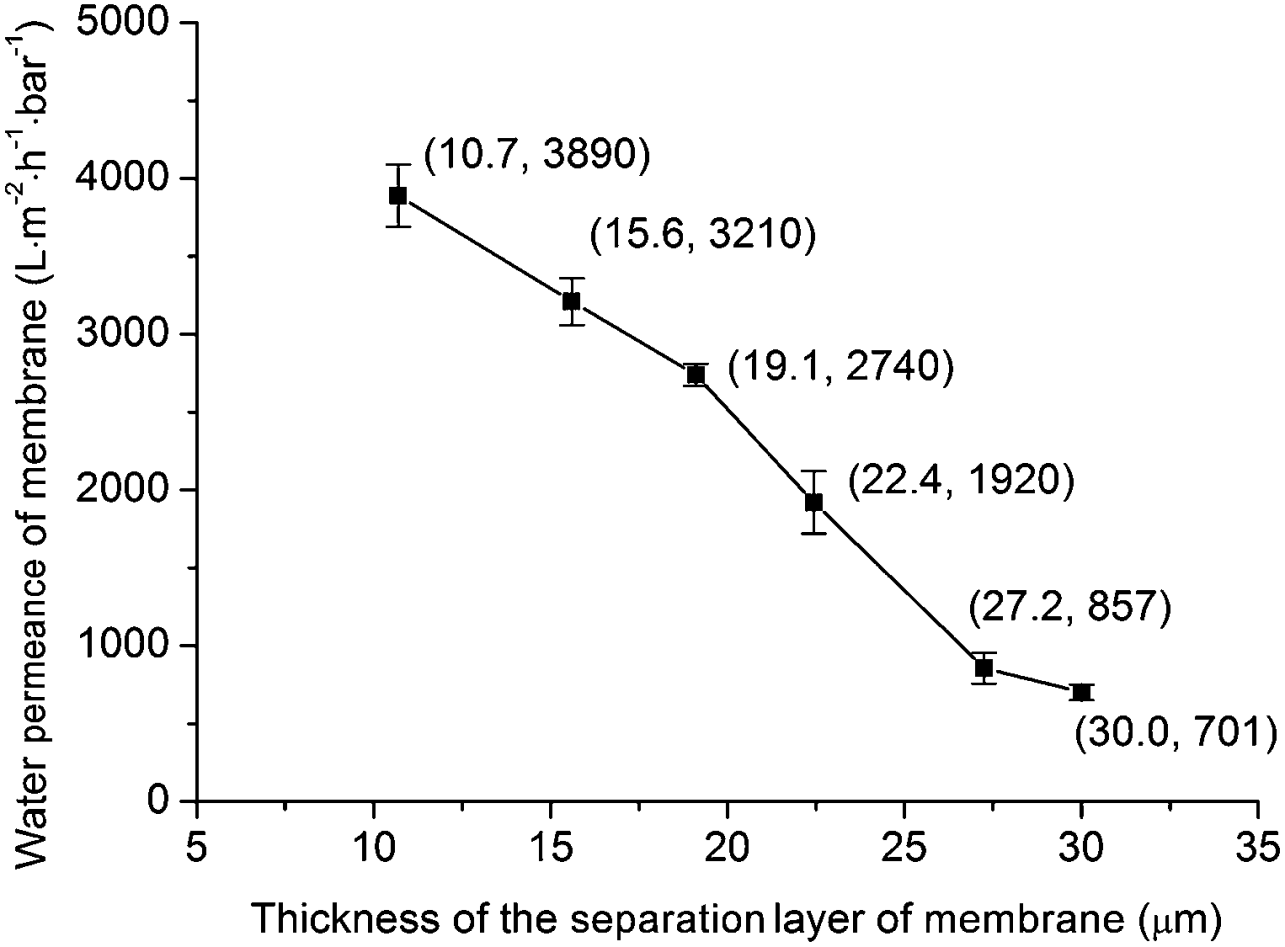
Water permeance of membrane *versus* thickness of the separation layer of membrane.

Fig. 13 shows the calculated water permeance of the membrane with different separation layer thickness (Δ*P* = 1 bar). It can be seen that with increasing the separation layer thickness from 10.7 μm to 30.0 μm, the water permeance of the membrane decreases from 3890 L m^−2^ h^−1^ bar^−1^ to 701 L m^−2^ h^−1^ bar^−1^. This trend is also similar to the Darcy's law for the flow through a porous material (eqn (2)): the water permeance of the membrane (*J*/Δ*P*) is reciprocal to the membrane thickness (*L*).

Table 3 lists the permeance of membranes for microfiltration in this work and literature. The average pore sizes of these membranes are at the same order of magnitude (0.1–0.5 μm). The membrane in this work shows relatively high water permeance in comparison with other reports. For example, for the membrane with average pore size = 0.18 μm and separation layer thickness = 10.7 μm, the water permeance arrives at 3890 L m^−2^ h^−1^ bar^−1^. While Qin *et al.*^16^ reported that for a membrane with average pore size = 0.24 μm and separation layer thickness = 10 μm, the water permeance was 1410 L m^−2^ h^−1^ bar^−1^. In Dong *et al.*,^8^ the membrane consisted of three layers: support layer, intermediate layer and separation layer. The average pore size of the intermediate layer was 0.85 μm which was close to the average pore size of the separation layer (0.54 μm). This intermediate layer reduced the water permeance of the membrane system. For the membrane systems in Wang *et al.*^10^ and Qin *et al.*,^16^ intermediate layer was avoided. However because dip-coating process was used in Wang *et al.*^10^ and Qin *et al.*,^16^ the water permeance might be reduced. For example, in Wang *et al.*^10^ the size of the titania powder for fabricating separation layer was 0.37 μm, and the average pore size of the support layer was 2.1 μm. The small titania powder would probably penetrate to the big pores of the support layer during the dip-coating process. In the proposed precursor film firing method, the membrane system was obtained without intermediate layer. What's more, because the dip-coating process was avoided the small grains in the separation layer didn't access to the big pores of the support. Because of above two reasons, the obtained membrane system shows relatively high water permeance.

**Table 3:** Water permeance of membranes in this work and literature

Membrane category	Average pore size (μm)	Separation layer thickness (μm)	Water permeance (L m^−2^ h^−1^ bar^−1^)	Reference
α-Al_2_O_3_	0.18	10.7	3.89 × 10^3^	This work
Zeolite	0.54	22.8	3.20 × 10^3^	Dong *et al.*^8^
TiO_2_	0.10–0.12	15–20	0.74 × 10^3^	Wang *et al.*^10^
α-Al_2_O_3_	0.24	10.0	1.14 × 10^3^	Qin *et al.*^16^

Table 4 lists the properties of support and separation layer. It is noted the porosity of the serration layer was not measured in this study.

**Table 4:** Comparison of the properties of support and separation layer

Properties	Support	Separation layer
Average pore size (μm)	4.6	0.18
Porosity	0.31	—
Permeance (L m^−2^ h^−1^ bar^−1^)	17.9 × 10^3^	3.89 × 10^3^

## 4. Conclusions

This study proposes a novel method to fabricate ceramic microfiltration membranes. A precursor film is prepared independently of the supports by a film coating machine. This precursor film consists of two layers: Al_2_O_3_/PVA layer and PVB layer. The precursor film is pasted on the support and fired to obtain the microfiltration membrane. Based on the experimental results, the following conclusion can be drawn:

The precursor film (Al_2_O_3_/PVA and PVB composite film) is flexible, which provides a potential for applying the precursor film firing method in fabricating curved membranes *e.g.* cylindrical membranes in addition to flat membranes.

The separation layer thickness (*L*_s_, μm) and the thickness of the Al_2_O_3_/PVA layer (*L*_p_, μm) follow the linear relationship: *L*_s_ = 0.76*L*_p_. This shows that it is convenient to control the thickness of the separation layer by using the precursor film firing method. With increasing the Al_2_O_3_/PVA layer thickness from 20 μm to 40 μm, the separation layer thickness increases from 10.7 μm to 30 μm. The separation layer of commercial microfiltration membranes is in normally in the range of 10–20 μm.^5^ In other words, the thickness of the separation layer fabricated with the precursor film firing method meets the commercial requirement.

Because intermediate layer and dip-coating process are avoided in the precursor film firing method, the fabricated membrane shows high permeance. For the fabricated membrane with average pore size = 0.18 μm and separation layer thickness = 10.7 μm, the water permeance reaches 3890 L m^−2^ h^−1^ bar^−1^.

In view of the above advantages, the proposed precursor film firing method is attractive for fabricating ceramic microfiltration membranes.

## Conflicts of interest

There are no conflicts to declare.

## Supplementary Material
